# Mass Spectrometry-Based Comparative Sequence Analysis for the Genetic Monitoring of Influenza A(H1N1)pdm09 Virus

**DOI:** 10.1371/journal.pone.0092970

**Published:** 2014-04-03

**Authors:** Jairo Gooskens, Jessika C. Zevenhoven-Dobbe, Eric C. Claas, Aloys C. M. Kroes, Clara C. Posthuma

**Affiliations:** Department of Medical Microbiology, Leiden University Medical Center, Leiden, The Netherlands; Johns Hopkins University - Bloomberg School of Public Health, United States of America

## Abstract

The pandemic influenza A (H1N1) 2009 virus (pH1N1) contains novel gene segments of zoonotic origin that lack virulence and antiviral resistance markers. We aimed to evaluate the applicability and accuracy of mass spectrometry-based comparative sequence analysis (MSCSA) to detect genetic mutations associated with increased virulence or antiviral resistance in pH1N1. During the 2009 H1N1 pandemic, routine surveillance specimens and clinical antiviral resistance monitoring specimens were analyzed. Routine surveillance specimens obtained from 70 patients with pH1N1 infection were evaluated for mutations associated with increased virulence (PB1-F2, PB2 and NS1 genes) or antiviral resistance (neuraminidase gene, NA) using MSCSA and Sanger sequencing. MSCSA and Sanger sequencing results revealed a high concordance (nucleotides >99%, SNPs ∼94%). Virulence or resistance markers were not detected in routine surveillance specimens: all identified SNPs encoded for silent mutations or non-relevant amino acid substitutions. In a second study population, the presence of H275Y oseltamivir resistant virus was identified by real-time PCR in 19 of 35 clinical antiviral resistance monitoring specimens obtained from 4 immunocompromised patients with ≥14 days prolonged pH1N1 excretion. MSCSA detected H275Y in 24% (4/19) of positive specimens and Sanger sequencing in 89% (17/19). MSCSA only detected H275Y when the mutation was dominant in the analyzed specimens. In conclusion, MSCSA may be used as a rapid screening tool during molecular surveillance of pH1N1. The low sensitivity for the detection of H275Y mutation in mixed viral populations suggests that MSCSA is not suitable for antiviral resistance monitoring in the clinical setting.

## Introduction

Influenza A and B viruses cause seasonal epidemics of febrile respiratory illness in the human population. The negative-sense RNA genome of influenza A viruses consists of eight segments which encode at least 13 proteins following transcription and translation [1]–[3]. Seasonal epidemics arise by antigenic drift through frequent mutations in hemagglutinin (HA) and neuraminidase (NA) surface glycoprotein genes enabling seasonal host immune evasion by the virus. In rare occasions, human influenza A virus gene segments reassort with other influenza A viruses from avian, human, and swine origins which can result in new antigenic subtypes that may cause severe human influenza pandemics [4].

In 2009, the influenza A (H1N1)pdm09 virus (pH1N1) with a relatively new antigenic subtype of zoonotic-origin emerged in Mexico and caused an influenza pandemic followed by rapid global spread in the human population [5]–[7]. The virus was likely generated through multiple reassortment events in pigs, and contained gene segments from North American “classical” swine influenza virus A/H1N1 (HA, NP, NS), North American avian influenza virus A/H1N1 (PB2, PA), human seasonal influenza virus A/H3N2 (PB1) and Eurasian swine influenza virus A/H1N1 (M, NA) [8]. The overall clinical presentation in humans appeared unexpectedly mild which can not be explained by residual host immunity in the human population. A minority of children and adults had preexisting cross-protective antibody titers against pH1N1 [9]. Only ∼18% of influenza A CD8+ cytotoxic T lymphocyte epitopes derived from previous seasonal influenza A viruses are conserved in pH1N1 [10]. The novel pH1N1 virus contained gene segments of zoonotic origin that lack virulence and antiviral resistance markers due to favorable genetic polymorphisms or truncations in PB2, PB1-F2, NS1 and NA genes [11]–[14].

Novel adaptive mutations may emerge in PB2, PB1-F2 and NS1 genes with the potential to increase viral replication and host pathogenicity of pH1N1 [14]–[16]. In addition, oseltamivir resistant virus may emerge during selective pressure [17], [18]. Wide-scale molecular surveillance is warranted but conventional sequencing methods are laborious and inefficient. Mass spectrometry-based comparative sequence analysis (MSCSA) enables rapid multigenomic sequence analysis with automated data analysis and could improve the availability of relevant pH1N1 genomic sequences [19], [20]. Rapid identification of relevant pH1N1 mutations in the community or in clinical settings may guide early prevention and intervention strategies. MSCSA was previously successfully used for the analysis of human cytomegalovirus sequence polymorphisms in UL97 gene and for detection of genetic mutations associated with antiviral resistance in clinical specimens [19].

In this study, we evaluated the applicability and accuracy of MSCSA to detect pH1N1 genetic mutations associated with increased virulence (PB1-F2, PB2 and NS1 genes) or antiviral resistance (NA gene).

## Materials and Methods

### Active Case Finding and Ethics Statement

**Figure 1:**
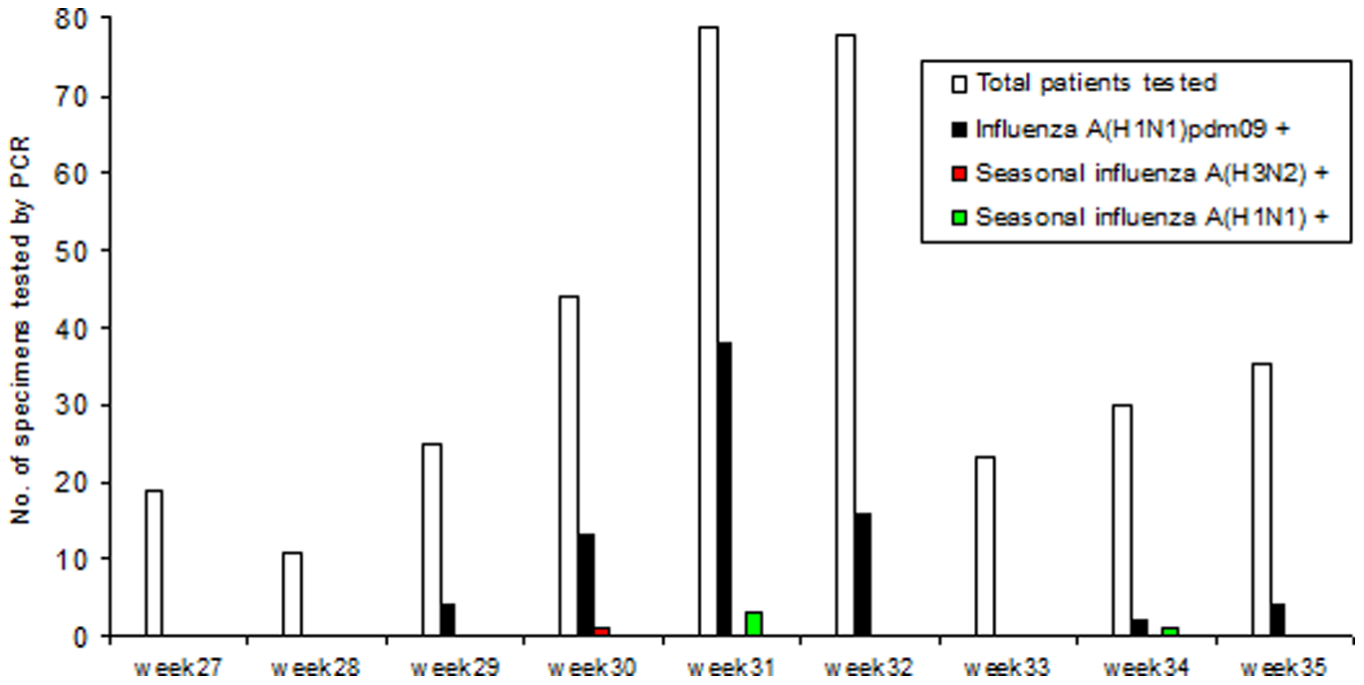
A graphic representation of the first wave of the 2009 H1N1 pandemic in the region of Leiden (The Netherlands).

During the 2009 H1N1 pandemic, human pH1N1 infection cases became obligatory notifiable by law in the Netherlands on 29 April 2009 (RIVM website. Available: www.rivm.nl/bibliotheek/rapporten/215011006.pdf. Accessed 2013 Mar 3.). A nationwide case definition was determined based on the European Union case definition [21] and contained clinical and epidemiological criteria upon which routine diagnostic testing was indicated and subsequent control measures could be applied. Diagnostic specimens were obtained from suspect cases by municipal health services, general practitioners or hospitals following influenza pandemic procedures from the National Institute of Public Health and the Environment (RIVM) responsible for the control of infectious diseases. In the region of Leiden, the first pandemic wave (weeks 27 through 35) inferred a normal (Gaussian) distribution of which the peak occurred in week 31 when 72% of positive patients were observed (Figure 1). All specimens included in this study were collected in the region of Leiden for routine influenza diagnostic testing following national pandemic procedures. The specimens were sent directly to the regional reference outbreak assistance laboratory accompanied by a written diagnostic request from the responsible phycisians and results were reported back to the medical facility who requested them. After performing routine influenza diagnostic testing, the reference outbreak assistance laboratory evaluated the additional presence of virulence and resistance markers for technical and epidemiological reasons. Additional influenza molecular characterization was not considered to be fully part of the initial diagnostic request, therefore all specimens were anonymized for precautionary reasons after receiving them from the requesting medical facilities and phycisians. There was no access to patient identifying information or clinical data during additional molecular characterization following ethical principles expressed in the Declaration of Helsinki. Approval from the institutional review board and the use of informed consents were not necessary for influenza diagnostic testing on specimens sent to the laboratory for this specific purpose.

### Study Populations

Two study populations with pH1N1 infection are under evaluation in this study. Routine surveillance specimens were obtained from 344 suspect cases in the region of Leiden during the early phase of the 2009 H1N1 pandemic (June 29^th^ through August 30^th^, 2009) and submitted to the regional reference outbreak assistance laboratory at Leiden University Medical Center. Influenza A virus was detected in 75 specimens (70 pH1N1, 4 seasonal H1N1, 1 seasonal H3N2). A total of 70 specimens from 70 patients with pH1N1 infection (43 male; median age 21 years, range 1 to 63 years) were included in this study. A second collection of 35 clinical antiviral resistance monitoring specimens was obtained from hospitalized immunocompromised patients with ≥14 days prolonged pH1N1 excretion (panH1 Ct values ≤ 37.0) during widespread pH1N1 circulation (November 3^rd^ 2009 through Janurary 13^th^, 2010). Real-time PCR confirmed H275Y mutated virus in 19 of 35 specimens obtained from 3 of 4 immunocompromised patients and these results were compared to MSCSA and Sanger sequencing findings of the NA gene.

### Influenza A Virus Detection and Molecular Typing

RNA was extracted from clinical specimens using the automated MagNA Pure nucleic acid isolation system (Roche Diagnostics, Almere, The Netherlands). Influenza A virus screening was performed by real-time PCR using InfA primers and a probe targeting the matrix gene and following procedures validated by the World Health Organization (WHO) and Centers for Disease Control and Prevention (CDC) as described previously (WHO website. Available: http://www.who.int/csr/resources/publications/swineflu/CDCrealtimeRTPCRprotocol_20090428.pdf. Accessed 2013 Mar 3.). The presence of pH1N1 was confirmed by PCR using panH1 and MexFluN1 protocols targeting the HA and NA genes [22], [23]. Influenza A virus specimens that were negative for pH1N1 were evaluated for seasonal influenza A (H1N1) and A (H3N2) viruses using H1-RF and H3-RF protocols targeting the HA gene [24] (WHO website. Available: http://www.who.int/csr/resources/publications/swineflu/WHO_Diagnostic_RecommendationsH1N1_20090521.pdf. Accessed 2013 Mar 3.).

### MSCSA, Sanger Sequencing and H275Y Real-time PCR

MSCSA and Sanger sequencing were performed on all specimen collections containing influenza A virus, whereas H275Y real-time PCR was only carried out on the clinical antiviral resistance monitoring specimens after which H275Y positive findings were correlated to MSCSA and Sanger sequencing results. H275Y real-time PCR was performed as described previously [22]. MSCSA using the MassARRAY/iSEQ™ – Comparative Sequence Analysis technique (Sequenom, San Diego, USA) is based on the PCR amplification of a genomic target and *in vitro* transcription to produce RNA strands. These transcripts are cleaved to produce a sequence-specific set of fragments for analysis by mass spectrometry [20]. As the MSCSA protocol starts from DNA, a reverse transcription reaction using SuperScriptII (Invitrogen) with random primers (Promega; 1 μg final concentration; incubations at 25°C for 10 min, at 40°C for 90 min and 70°C for 15 min) was implemented prior to PCR amplification. We designed T7 promoter-tagged (CAGTAATACGACTCACTATAGGGAGAAGGCT) forward primers and SP6-tagged (CGATTTAGGTGACACTATAGAAGAGAGGCT) reverse primers for the specific amplification of NA, PB1-F2, PB2 and NS1 targets (Table 1). The pH1N1 primers were designed to cover potential markers of virulence (PB2 gene positions 271, 590, 591, 627, 701; PB1-F2 gene position 66; NS1 gene positions 227–230), protein segment truncation (PB1-F2 gene positions 12, 58, 88; NS1 gene position 220) and antiviral resistance (NA gene positions 116, 117, 119, 136, 150, 151, 199, 223, 275 and 295) in the viral genome (Table 2) [11]–[14], [25], [26]. PCR amplification of the targets was performed using 2 μl of RT product, with the tagged primers in 10 μl volume in 384 well plates as previously described [19]. The sample was processed by shrimp alkaline phosphatase treatment, *in vitro* transcription, and C- or U-specific RNaseA cleavage, according to the manufacturer’s instructions and using a MassARRAY Liquid Handler (Matrix+Fusio™ Chip Module, Sequenom). The fragments resulting from RNA cleavage were diluted in double-distilled water and desalted with Clean Resin (Sequenom), transferred to a SpectroCHIP array (Sequenom), and analyzed by matrix-assisted laser desorption ionization time-of-flight (MALDI-TOF) mass spectrometry (MassARRAY Compact Analyzer, Sequenom). Sanger sequencing was done on 2 μl of the PCR amplified products that were produced during the MSCSA procedure. Sanger sequencing reactions were performed in a volume of 10 μl using Big Dye terminator v 1.1 with sequencing buffer (Applied Biosystems) and 1 μM primer. The primers for Sanger sequencing were identical to the primers used for MSCSA (Table 1), but without the T7- and SP6-containing tails. Nucleotide sequence analysis was performed on an ABI Prism 3100 Genetic Analyser (Applied Biosystems).

**Table 1:** Overview of pH1N1 Sanger sequencing and MSCSA primers and amplicon sizes.

Gene	Forward primer	Oligonucleotide sequence	Reverse primer	Oligonucleotide sequence	Amplicon size
NA	Flu-NA_F (274)$	tgccctgttagtggatggc	Flu-NA_SP6R (988)#	cattagggcgtggattgtctc	715 Bp
PB1-F2	Flu-PB1-F2_F (1)$	atggatgtcaatccgactctac	Flu-PB1-F2_SP6R (476)#	tcattagctgttaggccattcg	476 Bp
PB2	Flu-PB2_F (1764)$	cagaagccggtacagtggattc	Flu-PB2_SP6R (2201)#	accaacactacgtccccttgc	438 Bp
NS1	Flu-NS1_F (211)$	cttgaaagaggaatccagcgag	Flu-NS1_SP6R (740)#	caatctgtgccgcatttcttc	530 Bp

Bp, base pairs.

$ 5′ position of the first nucleotide of the forward primer in the corresponding gene;

# 5′ position of the last nucleotide of the reverse primer in the corresponding gene.

**Table 2:** Surveillance of pH1N1 genetic markers associated with virulence or antiviral resistance.

Gene	Antiviral resistance	Increased virulence	Protein truncation
NA	V116, I117, E119, Q136, K150, D151, D199, I223, H275, N295	none	none
PB1-F2	none	N66	Stop12, Stop58, Stop88
PB2	none	A271, S590, R591, E627, D701	none
NS1	none	G227, T228, E229, I230	Stop220

Relevant amino acid genetic positions are depicted for each corresponding gene.

### Data Analysis

For MSCSA, the mass spectra of four amplicon transcript cleavage products per sample were matched against cleavage patterns calculated from an imported set of reference sequences [20]. The reference database was created by the authors and included a set of 193 NA gene, 67 PB2 gene, 210 NS1 gene and 53 PB1-F2 gene sequences imported from a resource database in September 2009 (PubMed website. Available: http://www.ncbi.nlm.nih.gov/genomes/FLU/FLU.html. Accessed 2009 Sep 3.). Data processing was performed using iSEQ Software Version 1.0.0.2 to assemble amplicon sequences. MSCSA and Sanger sequences were aligned to influenza A/california/04/2009 (H1N1) and single nucleotide polymorphisms (SNPs) were defined as nucleotide variations to this reference sequence.

## Results

### MSCSA Primers and Amplicons

Primers utilized for MSCSA were specific for pH1N1 as they did not amplify seasonal influenza A(H1N1 or H3N2). Among 70 routine surveillance specimens, the primers amplified 265 of in total 280 amplicons (95%) including NA (63/70), PB1-F2 (65/70), PB2 (68/70) and NS1 genes (69/70). PCR amplification of PB1-F2, PB2 and NS1 genes only failed in specimens with low viral levels (mean InfA PCR Ct 36.2), whereas amplification was observed in specimens containing high viral levels (mean InfA PCR Ct 26.5). The amplification of the NA gene failed in 4 specimens with high viral levels (range Ct 22.3 to 28.1), suggesting that occasionally the primer sequence did not correspond to the actual viral sequence.

### MSCSA and Sanger Sequencing of Routine Surveillance Specimens

Using the resource database in September 2009 as reference database for MSCSA, 99.9% of the total number of nucleotides (n = 130204) matched between both methods. A total of 93.6% (456/487) of all SNPs were identified by both methods (Table 3). More accurate results were obtained for the NA, PB2 and NS1 amplicons (93.2%, 98.7% and 90.1%, respectively) compared to the PB1-F2 amplicon (82.9%). In a small number of cases, a SNP detected by both methods could not be pinpointed to an exact position within an eleven nucleotide region by MSCSA (17/487, 3.5%). These inconclusive sequence data resulted in <0.1% uncertain nucleotide positions and were not considered as mismatches. A high degree of concordance was observed for the detection of SNPs via MSCSA and Sanger sequencing. The majority of SNPs (246/456) resulted in silent mutations and few SNPs (210/456) encoded for non-relevant amino acid substitutions in NA gene (V106I (n = 61), V203M (n = 1), N248D (n = 62), S286G (n = 1)), PB1-F2 gene (T34A (n = 1), V113A(n = 1)), PB2 gene (K660R (n = 2)) and NS1 gene segments (I123V (n = 61), N133D (n = 16), S135N (n = 1), G154R (n = 1), V194I (n = 1), D207N (n = 1)). The degree of discordance was limited (31/487 SNPs) and only 8 amino acid substitutions differed among the results generated by MSCSA and Sanger sequencing. We detected no mutations associated with increased virulence or with reversion of protein segment truncation by either method. All surveillance specimens had wild-type sequences in virulence markers (PB2 gene A271, S590, R591, E627, D701; PB1-F2 gene N66; NS1 gene G227, T228, E229 and I230) and stop codons in protein truncation markers (PB1-F2 stop12, stop58, stop88; NS1 gene stop220). MSCSA and Sanger sequencing detected no mutations associated with antiviral resistance (NA gene V116, I117, E119, Q136, K150, D151, D199, I223, H275 and N295).

**Table 3:** MSCSA and Sanger sequencing of 70 pH1N1 virus specimens.

Target	Evaluation	Total	NA gene	PB1-F2 gene	PB2 gene	NS1 gene
Nucleotides	Genetic sequence	130204	42525	27216	26860	33603
	Match (%)#	130173 (99,98%)	42512 (99,97%)	27210 (99,98%)	26858 (99,99%)	33593 (99,97%)
Amplicons	Genetic sequence	263	63	63$	68	69
	Match (%)#	239 (90,9%)	54 (85,7%)	58 (92,1%)	66 (97,1%)	61 (88,4%)
SNPs	Genetic sequence	487	192	35	154	106
	Match (%)#	456 (93,6%)	179 (93,2%)	29 (82,9%)	152 (98,7%)	96 (90,1%)
	SNPs in MSCSA^1^	13	8	0	0	5
	SNPs in Sanger^2^	18	5	6	2	5

$ Sanger sequencing was succesful in 63 of 65 specimens determined by MSCSA.

# Sequence match using MSCSA and Sanger sequencing.

1 SNPs in MSCSA and not in Sanger sequencing;

2 SNPs in Sanger and not MSCSA.

### Clinical Antiviral Resistance Monitoring Specimens

Oseltamivir resistant H275Y mutated virus was confirmed in 19 of 35 specimens clinical antiviral resistance monitoring specimens by H275Y real-time PCR and these findings were compared to MSCSA and Sanger sequencing results. MSCSA detected NA gene H275Y mutation in 4 of 19 samples (24%) while the mutation was observed in 17/19 samples by Sanger sequencing (89%). MSCSA detected NA gene H275Y mutation in specimens with fully mutant virus populations (4/4) but not in specimens with mixed wildtype and mutant populations (0/15). By visual evaluation of NA gene mass spectrometry spectra, the presence of peaks derived from the wild type sequence as well as from the H275Y-associated mutation were observed in many samples. In this way, we were able to infer the presence of H275Y minor populations in 14 pH1N1 samples, which led to a 95% correspondence between MSCSA and real-time PCR (18 of 19 samples).

## Discussion

Molecular surveillance of human influenza viruses is important to monitor viral evolution. Genetic mutations occur frequently due to the lack of proofreading by the influenza virus RNA polymerase and may emerge during recurrent interspecies transmission or wide-scale antiviral selective pressure [1], [7], [14]–[17]. Rapid identification of relevant mutations associated with increased virulence or antiviral resistance during active surveillance of pH1N1 may guide early prevention and treatment strategies. MSCSA enables medium- to high-throughput genetic sequence analysis of amplified targets of genomic DNA or RNA with automated data analysis [20]. During the 2009 H1N1 pandemic, we evaluated influenza A-positive routine surveillance specimens for virulence or resistance markers using MSCSA and Sanger sequencing and included H275Y real-time PCR on clinical antiviral resistance monitoring specimens. MSCSA did not cover the complete pH1N1 genome since we chose to focus on gene segments (Table 2) that contain relevant virulence or resistance markers (with the exception of the HA gene) [11]–[14], [25], [26].

We observed a high concordance between MSCSA and Sanger sequencing (nucleotides >99%, SNPs 93.6%) in 70 routine surveillance specimens and detected 487 SNPs. Most SNPs encoded for silent mutations and few encoded for non-relevant amino acid changes compared to reference influenza A/california/04/2009 (H1N1) virus. The most frequently detected non-relevant amino acid changes in the NA gene (V106I (n = 61), N248D (n = 62)) and NS1 gene segments (I123V (n = 61), N133D (n = 16)) are well described by others [27]. Genetic mutations associated with increased virulence (PB2 gene positions 271, 590, 591, 627, 701; PB1-F2 gene position 66; NS1 gene positions 227–230), reversion of protein segment truncation (PB1-F2 gene positions 12, 58, 88; NS1 gene position 220) or antiviral resistance (NA gene positions V116, I117, E119, Q136, K150, D151, D199, I223, H275 and N295) were not detected by either method in routine surveillance specimens [11]–[14], [25], [26]. Amino acid replacement at residue S334 associated with increased oseltamivir resistance was not monitored in the NA amplicon. This is not of great concern because mutations at amino acid S334 can only increase oseltamivir resistance in the presence of H275Y mutation but does not lead to phenotypic antiviral resistance by itself [26]. Genetic sequences of pH1N1 circulating in the region of Leiden appeared genetically stable, similar to pH1N1 during the early phase of the 2009 H1N1 pandemic in Mexico and the United States [7]. MSCSA could not accurately pinpoint the exact position of a small number of SNPs in spite of the reference database. Relevant SNPs detected by MSCSA always need to be confirmed by Sanger sequencing, pyrosequencing or real-time PCR to limit potential misinterpretations during molecular surveillance. We emphasize that the reference database must be continuously updated with recent virus sequences for MSCSA to remain accurate. The MSCSA reference database has not been updated since the 2009 H1N1 pandemic, therefore newer versions of the reference database were not available to re-analyze the data.

Influenza reverse transcription-polymerase chain reaction/electro-spray ionization (RT-PCR/ESI-MS) assay is a similar mass spectrometry-based automated method which is capable of measuring amplicon-derived masses. The benefit of MSCSA over RT-PCR/ESI-MS is the possibility to analyze larger amplicon sizes (500–800 versus 150 nucleotides) and its capability to detect all nucleotide variations within the analyzed target region rather than providing genomic signatures [28], [29]. Some methods for antiviral resistance detection, like real-time PCR, are fast, but only allow for the analysis of fixed genome positions known to be involved in antiviral drug resistance.

In this study, a second collection of clinical antiviral resistance monitoring specimens was included and real-time PCR detected oseltamivir resistant H275Y mutated virus in 19 of 35 specimens obtained from 3 of 4 immunocompromised patients. We detected no other mutations that are associated with antiviral resistance including NA gene positions V116, I117, E119, Q136, K150, D151, D199, I223 and N295 [25], [26]. Previous influenza studies have shown that immunocompromised patients with prolonged viral excretion are at increased risk for developing neuraminidase inhibitor resistant virus during continued oseltamivir treatment [30], [31]. Frequent development of antiviral resistant viruses among 3 of 4 (75%) immunocompromised patients is in agreement with previous studies [32]–[34]. MSCSA detected H275Y in 24% (4/19) of positive specimens and Sanger sequencing in 89% (17/19). MSCSA only detected H275Y when the mutation was dominant in the analyzed specimens. The ability to infer the presence of H275Y (18 of 19 samples) by visual evaluation of the mass spectrometry spectra indicated that iSEQ software improvement may result in improved detection of minor variants in mixed populations. However, pyrosequencing and real-time PCR currently remain the designated methods for continued antiviral resistance monitoring in clinical settings [22], [25].

In conclusion, MSCSA may be used as a rapid screening tool to monitor fixed nucleotide changes and potential virulence markers in the pH1N1 genetic background. MSCSA does not seem applicable for continued antiviral resistance monitoring in the clinical setting since detection of the H275Y mutation was limited by a very low sensitivity in the presence of minor variants and mixed genotypes.
